# Cell‐of‐origin determined by both gene expression profiling and immunohistochemistry is the strongest predictor of survival in patients with diffuse large B‐cell lymphoma

**DOI:** 10.1002/ajh.25666

**Published:** 2019-11-07

**Authors:** Maysaa Abdulla, Peter Hollander, Tatjana Pandzic, Larry Mansouri, Susanne Bram Ednersson, Per‐Ola Andersson, Magnus Hultdin, Maja Fors, Martin Erlanson, Sofie Degerman, Helga Munch Petersen, Fazila Asmar, Kirsten Grønbæk, Gunilla Enblad, Lucia Cavelier, Richard Rosenquist, Rose‐Marie Amini

**Affiliations:** ^1^ Clinical and Experimental Pathology, Department of Immunology, Genetics and Pathology Uppsala University Uppsala Sweden; ^2^ Medical Genetics and Genomics, Department of Immunology, Genetics and Pathology Uppsala University Uppsala Sweden; ^3^ Department of Molecular Medicine and Surgery Karolinska Institute Stockholm Sweden; ^4^ Department of Pathology Sahlgrenska University Hospital Gothenburg Sweden; ^5^ Sahlgrenska Academy at the University of Gothenburg Gothenburg Sweden; ^6^ Department of Medicine Södra Älvsborg Hospital Borås Borås Sweden; ^7^ Department of Medical Biosciences Pathology, Umeå University Umeå Sweden; ^8^ Department of Radiation Sciences, Oncology Umeå University Umeå Sweden; ^9^ Department of Pathology Copenhagen University Hospital, Rigshospitalet Copenhagen Denmark; ^10^ Department of Hematology Copenhagen University Hospital, Rigshospitalet Copenhagen Denmark; ^11^ Experimental and Clinical Oncology, Department of Immunology, Genetics and Pathology Uppsala University Uppsala Sweden

## Abstract

The tumor cells in diffuse large B‐cell lymphomas (DLBCL) are considered to originate from germinal center derived B‐cells (GCB) or activated B‐cells (ABC). Gene expression profiling (GEP) is preferably used to determine the cell of origin (COO). However, GEP is not widely applied in clinical practice and consequently, several algorithms based on immunohistochemistry (IHC) have been developed. Our aim was to evaluate the concordance of COO assignment between the Lymph2Cx GEP assay and the IHC‐based Hans algorithm, to decide which model is the best survival predictor. Both GEP and IHC were performed in 359 homogenously treated Swedish and Danish DLBCL patients, in a retrospective multicenter cohort. The overall concordance between GEP and IHC algorithm was 72%; GEP classified 85% of cases assigned as GCB by IHC, as GCB, while 58% classified as non‐GCB by IHC, were categorized as ABC by GEP. There were significant survival differences (overall survival and progression‐free survival) if cases were classified by GEP, whereas if cases were categorized by IHC only progression‐free survival differed significantly. Importantly, patients assigned as non‐GCB/ABC *both* by IHC and GEP had the worst prognosis, which was also significant in multivariate analyses. Double expression of MYC and BCL2 was more common in ABC cases and was associated with a dismal outcome. In conclusion, to determine COO *both* by IHC and GEP is the strongest outcome predictor to identify DLBCL patients with the worst outcome.

## 1 INTRODUCTION

Diffuse large B‐cell lymphoma (DLBCL) is the most common form of aggressive lymphoma and is a heterogeneous disease with different histopathologic, phenotypic and genetic features with varying clinical outcomes.^1^, ^2^ Based on gene expression profiling (GEP), the tumor cells are considered to be derived from activated B‐cells (ABC) or germinal center B‐cells (GCB).^3^, ^4^ Several studies have shown a survival benefit for DLBCL patients with a GCB phenotype compared to an ABC phenotype,^5^, ^6^ whereas other have not.^7^, ^8^, ^9^ In addition, a third group with unclassified cases (UC) was reported, and proposed to have an inferior outcome similar to ABC‐DLBCL.^4^, ^10^

In the updated World Health Organization (WHO) classification of Tumors of Hematopoietic and Lymphoid Tissues,^2^ information on the cell‐of‐origin (COO), either by immunohistochemical (IHC) stainings^6^, ^11^, ^12^, ^13^ or by GEP, is required for a definite DLBCL diagnosis. In clinical practice, however, the use of GEP has not been widely adopted. Most GEP technologies require fresh‐frozen tumor tissue. But in the daily, clinical diagnostic work‐up, formalin fixed, paraffin‐embedded (FFPE) tissue is the primary source, and fresh‐frozen material is not routinely collected. Therefore, IHC algorithms have been developed as substitutes and applied with varying concordance to GEP. The most commonly used classification is the Hans algorithm^11^ based on the IHC staining results of three proteins: CD10, BCL6 and MUM1, although other systems have also been proposed.^6^, ^12^, ^13^ However, these IHC algorithms will only identify two groups; GCB or non‐GCB, since they cannot identify cases classified as UC by GEP. In recent years, the NanoString technology Lymph2Cx assay was developed based on GEP, which shows a strong concordance to the original COO model and can be applied on FFPE tissue.^10^, ^14^, ^15^, ^16^ This assay uses a limited set of 15 pre‐specified genes and five housekeeping genes, and has the potential to identify all three subgroups of DLBCL.^17^, ^18^ In this study, our aim was to investigate the concordance between the Lymph2Cx assay and the IHC algorithm by Hans et al.^11^ in relation to clinical characteristics, tumor markers and survival outcome. This was to identify which model would be the best survival predictor in a large cohort of Swedish and Danish DLBCL patients (n = 359).

## 2 METHODS

### 2.1 Patients

Three hundred and fifty‐nine patients were included in the study and diagnosed with de novo DLBCL between 2004‐2015 in Sweden and Denmark. Patients included had primary DLBCL of the central nervous system, and immunodeficiency‐associated lymphoproliferative disorders (PTLD). Included patients also had unclassifiable B‐cell lymphoma, with features intermediate between diffuse large B‐cell lymphoma and Burkitt lymphoma,. And, primary mediastinal B‐cell lymphomas were excluded but all other extranodal and nodal DLBCL were included. Cases were classified according to the 2008 WHO classification. All patients were homogeneously treated with R‐CHOP (rituximab, cyclophosphamide, doxorubicin, vincristine and prednisone) or R‐CHOP‐like regimens. Patients with a known previous history of a low‐grade lymphoma were excluded. Clinical information was collected from patient records. Patients were followed‐up with clinical examinations and radiologic examinations were used when relapse or progressive disease was suspected. Age‐adjusted International Prognostic Index (aaIPI) was used (one point for each: (a) Ann Arbor stage III‐IV; (b) elevated serum lactate dehydrogenase (LDH); (c) and ECOG performance status 2‐3). Here 0‐1 is considered to be low risk and 2‐3 is considered to be high risk, in accordance with national guidelines in Sweden and Denmark.

### 2.2 RNA extraction

Extraction of RNA from FFPE tissue was done according to the AllPrep DNA/RNA Mini Kit for FFPE protocol (Qiagen, Hilden, Germany). That protocol allows for the simultaneous purification of genomic DNA and total RNA from the same biological sample. Purification of RNA was done with the AllPrep column flow‐through, using an RNeasy Mini spin column.

### 2.3 NanoString assay

Samples were analyzed with the Lymph2CX assay on a NanoString instrument according to the manufacture's instructions. The dataset was analyzed using the research use only (RUO) version of the NanoString Lymphoma Subtyping Test (LST), which is based on the Lymph2Cx assay, to determine the COO molecular subtype of each sample.^19^ The LST algorithm measures the geometric mean of five housekeeping genes (HK geomean), to ensure RNA quality based on a pre‐defined clinical QC threshold of 128. An HK geomean value below 64 was deemed as insufficient RNA quality to provide a subtyping result. A value between 64 and 128 was considered to be borderline quality since it meets previously published thresholds for RNA quality within clinical research studies,^18^ but does not meet the clinical QC threshold of 128 for individual patients. Each sample surpassing the QC threshold was reported as one of the two molecular subtypes, ABC, GCB, or UC within an equivocal zone. Three hundred and eight cases (86%) passed, 44 failed (12%) and 7 (2%) were considered to be borderline. Laboratory work was carried out at Uppsala University Hospital according to SOP provided by Nanostring. Data was analyzed by Nanostring (we did not obtain the algorithm).

### 2.4 Immunohistochemical stainings

The IHC stainings for CD10, BCL2, BCL6, MUM1 and MYC were performed at the different sites according to routine procedures in each diagnostic laboratory. The stainings were re‐evaluated semi‐quantitatively by each site's hematopathologists (authors MH, MF, MA, SBE, HMP). The Hans algorithm was applied to classify tumors as GCB or non‐GCB by IHC, and included CD10, BCL6 and MUM1 stainings with a cut‐off of 30% positive tumor cells. For MYC, a cut‐off of 40% was applied and for BCL2 50%. Since insufficient material was a problem in a majority of the cases, FISH analyses for *BCL2* and *MYC* were not performed.

### 2.5 Cell‐of‐origin groups

The following subgroups were defined according to GEP or IHC:

1. ABC = ABC type defined by GEP and classified with the Lymph2Cx assay.
2. GCB‐GEP = GCB type defined by GEP and classified with the Lymph2Cx assay.
3. UC = unclassified cases defined by GEP and classified with the Lymph2Cx assay.
4. Non‐GCB = ABC type defined by IHC according to the Hans algorithm.
5. GCB‐IHC = GCB type defined by IHC according to the Hans algorithm.

Three different comparisons of COO analyzes according to the Hans algorithm and/or the Lymph2Cx assay are presented in the main manuscript: (a) ABC vs GCB‐GEP; (b) non‐GCB *vs* GCB‐IHC; (c) ABC and non‐GCB combined *vs* cases with information on both the Lymph2cx assay and the Hans algorithm, that were not ABC and non‐GCB combined. Additional COO groups were studied and are presented in the supplementary material (Supplementary methods Tables S1 and S2).

### 2.6 Ethics

The study was conducted in accordance with the Declaration of Helsinki and was approved by the Regional Ethical Committees in Sweden and Denmark (Dnr 233/2014, Dnr 198/2010, 140‐10, T753‐12, T216‐13, T316‐15, HD‐2009‐003).

### 2.7 Statistical analyses

Tabulated values were compared using the chi‐square or the Fisher's exact test. Student's *t*test was used to compare means between groups. Pearson's test was applied to determine correlative associations between parameters. Overall survival (OS) was calculated from the date of diagnosis to the date of death of any cause. Progression‐free survival (PFS) was calculated from the date of diagnosis to the date of lymphoma progression or death due to any cause. Survival curves and univariate analyses were performed using the Kaplan‐Meier method, and the log‐rank test and Cox proportional hazards regression were used to compare differences between groups. Cases with missing information on clinical or pathological variables were not included in the survival analyses. Multivariate Cox proportional hazards regression models included prognostic variables of at least borderline significance (*P* < .10). Cases with one or more missing variables were omitted from the multivariate analysis. The proportional hazards assumption was tested and was not violated. A *P* value <.05 was considered to be statistically significant. Statistical analyses were performed using RStudio 1.1.383 (http://www.r-project.org).

## 3 RESULTS

### 3.1 Comparison of COO according to the Lymph2Cx assay and the Hans algorithm

Three hundred and fifty‐one cases were investigated with IHC markers to determine COO according to the Hans algorithm, where 180 cases (51%) were classified as GCB‐IHC, and 171 cases (49%) as non‐GCB (Table S3). In total, 315 cases were successfully investigated with the NanoString Lymph2Cx assay to determine COO, whereas 44 cases failed to pass the analysis due to insufficient RNA quality. One hundred and sixty‐eight cases (53%) were classified as GCB‐GEP, 105 cases (33%) as ABC, and 42 cases (13%) as UC according to the Lymph2Cx assay.

Three hundred and eight cases had information on COO status according to both the Lymph2Cx assay and IHC with the Hans algorithm. Of 151 cases categorized as non‐GCB according to the Hans algorithm, 88 (58%) were grouped as ABC, 30 (20%) as GCB‐GEP and 33 (22%) as UC by the Lymph2Cx assay. Of 157 cases assigned as GCB‐IHC according to the Hans algorithm, 133 (85%) were classified as GCB‐GEP, 15 (9%) as ABC, and 9 (6%) as UC by the Lymph2Cx assay (Table S3). The overall concordance between the Lymph2Cx assay and the Hans algorithm to determine COO was 72%, and 83% when UC cases were excluded. In the latter case, a relatively high correlation between the Lymph2Cx assay and the Hans algorithm to determine COO was observed (Pearson's correlation coefficient = 0.66 [95% CI 0.58‐0.72], *P* < .001).

### 3.2 Clinicobiological correlations

In the entire cohort, there were 203 men and 154 women with a male to female ratio of 1.3:1. The median age at diagnosis was 66 years and the mean age was 64 years (range, 18‐89 years). One hundred and thirty‐two patients (37%) presented with B‐symptoms, 147 (41%) had ≥2 aaIPI risk factors and 107 (30%) presented with extranodal involvement (Table 1). The median follow‐up time for all patients was 70 months (range, 0.2‐188 months). Two patients were lost to follow‐up and excluded from the survival analyses. In 105 patients with ABC according to the Lymph2Cx assay, a higher proportion of cases were aged ≥60 years. They also had more often double expression of MYC and BCL2 compared with cases with GCB‐GEP (*P* = .01) (Table 1). In contrast, while a higher proportion of cases had B symptoms in 171 patients with non‐GCB according to the Hans algorithm, there was no statistically significant difference in double expression of MYC and BCL2 (*P* = .1), compared with cases with GCB‐IHC (Table 1). In 88 patients with ABC according to the Lymph2Cx assay and non‐GCB according to IHC, a higher proportion of cases was again aged ≥60 years and had more often double expression of MYC and BCL2, compared with cases that were not ABC and non‐GCB combined (*P* = .009) (Table 1).

**Table 1:** Clinicopathological variables and their distribution in the whole cohort, in patients with ABC and GCB‐GEP, non‐GCB and GCB‐IHC, and in patients with ABC *and* non‐GCB combined, and cases not classified as ABC and non‐GCB combined

	Whole cohort (%)	ABC (%)	GCB‐GEP (%)	*P* value^a^	Non‐GCB (%)	GCB‐IHC	*P* value^b^	ABC *and* non‐GCB combined (%)	*Not* ABC *and* non‐GCB combined (%)	*P* value^c^
All patients	359 (100)	105 (100)	168 (100)		171 (100)	180 (100)		88 (100)	220 (100)	
Age				**<.001** d			.44^d^			**.002** d
Mean	64	69	63		65	64		68	63	
Median	66	70	65		66	67		70	65	
Range	18‐89	34‐86	22‐89		25‐85	18‐89		34‐85	18‐89	
Age ≥ 60 years				**.003**			.58			**.04**
Yes	246 (69)	86 (82)	111 (66)		121 (71)	123 (68) 22257		71 (81)	148 (67)	
No	110 (31)	18 (17)	56 (33)		48 (28)	32		17 (19)	70 (32)	
Missing	3 (1)	1 (1)	1 (1)		2 (1)	0 (0)		0 (0)	2 (1)	
Male				.54			.97			.99
Yes	203 (57)	62 (59)	92 (55)		96 (56)	103 (57)		51 (58)	127 (58)	
No	154 (43)	42 (40)	75 (45)		74 (43)	77 (43)		37 (42)	92 (42)	
Missing	2 (1)	1 (1)	1 (1)		1 (1)	0 (0)		0 (0)	1 (0)	
B symptoms				.68			**.03**			.72
Yes	132 (37)	39 (37)	55 (33)		73 (43)	57 (32)		35 (40)	78 (35)	
No	207 (58)	62 (59)	101 (60)		88 (51)	114 (63)		50 (57)	127 (58)	
Missing	20 (6)	4 (4)	12 (7)		10 (6)	9 (5)		3 (3)	15 (7)	
≥2 age‐adjusted IPI				.21			.62			.71
Yes	147 (41)	48 (46)	64 (38)		73 (43)	73 (41)		40 (45)	94 (43)	
No	183 (51)	48 (46)	92 (55)		83 (49)	95 (53)		41 (47)	110 (50)	
Missing	29 (8)	9 (9)	12 (7)		15 (9)	12 (7)		7 (8)	16 (7)	
Stage ≥ III				.37			.63			.95
Yes	198 (55)	61 (58)	86 (51)		98 (57)	98 (54)		51 (58)	124 (56)	
No	140 (39)	38 (36)	70 (42)		63 (37)	72 (40)		32 (36)	82 (37)	
Missing	21 (6)	6 (6)	12 (7)		10 (6)	10 (6)		5 (6)	14 (6)	
High LDH				.92			.42			.99
Yes	184 (51)	55 (52)	84 (50)		92 (54)	89 (49)		47 (53)	117 (53)	
No	157 (44)	45 (43)	73 (43)		70 (41)	83 (46)		37 (42)	91 (41)	
Missing	18 (5)	5 (5)	11 (7)		9 (5)	8 (4)		4 (5)	12 (5)	
Extranodal involvement				.20			.84			.51
Yes	107 (30)	36 (34)	44 (26)		55 (32)	52 (29)		30 (34)	63 (29)	
No	167 (47)	45 (43)	83 (49)		82 (48)	84 (47)		40 (45)	106 (48)	
Missing	85 (24)	24 (23)	41 (24)		34 (20)	44 (24)		18 (20)	51 (23)	
High expression of MYC				.052			.86			**.04**
Yes	44 (12)	20 (12)	17 (16)		23 (13)	21 (12)		16 (18)	24 (11)	
No	117 (33)	60 (36)	21 (20)		57 (33)	59 (33)		21 (24)	77 (35)	
Missing	198 (55)	88 (52)	67 (64)		91 (53)	100 (56)		51 (58)	119 (54)	
High expression of BCL2				**.002**			**.03**			**.003**
Yes	151 (42)	62 (370	50 (48)		84 (49)	67 (37)		46 (52)	85 (39)	
No	61 (17)	38 (23)	8 (8)		23 (13)	37 (21)		6 (7)	45 (20)	
Missing	147 (41)	68 (40)	47 (45)		64 (37)	76 (42)		36 (41)	90 (41)	
Double expression of MYC and BCL2				**.01**			.12			**.009**
Yes	33 (9)	15 (14)	13 (8)		21 (12)	12 (7)		14 (16)	17 (8)	
no	136 (38)	25 (24)	72 (43)		63 (37)	72 (40)		23 (26)	91 (41)	
missing	190 (53)	65 (62)	83 (49)		87 (51)	96 (53)		51 (58)	112 (51)	

Abbreviations: ABC, activated B‐cell; GCB, germinal center‐derived B‐cell; IHC, immunohistochemical; GEP, gene expression profiling; IPI, International Prognostic Index; LDH, Lactate Dehydrogenase.

*Note*: Boldface font indicates statistical significance (*P* < 0.05).

a Comparing ABC vs GCB‐GEP.

b Comparing non‐GCB vs GCB‐IHC.

c Comparing ABC and non‐GCB according to both the Hans algorithm and the Lymph2Cx assay vs cases that were not ABC and non‐GCB combined.

d *P* value according to Student's *t* test.

### 3.3 Univariate survival analysis

**Figure 1:**
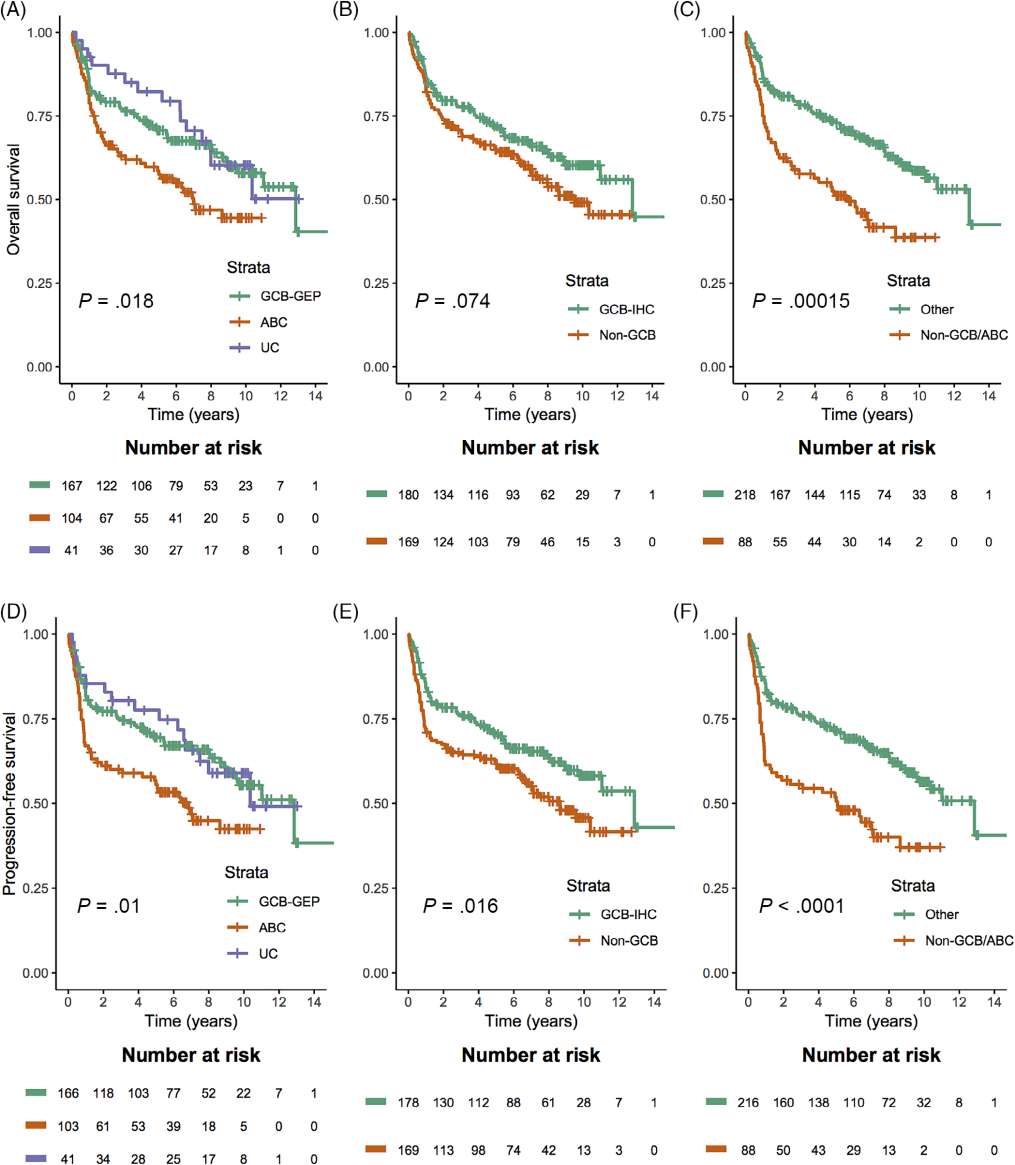
Kaplan–Meier curve for overall survival according to (A) the Lymph2Cx assay, (B) the Hans algorithm and (C) the Lymph2Cx assay and the Hans algorithm combined, and for progression‐free survival according to, (D) the Lymph2Cx assay, (E) the Hans algorithm, (F) the Lymph2Cx assay and the Hans algorithm combined

Patients classified as ABC according to the Lymph2Cx assay had significantly inferior five‐year survival rates at 58% for OS and 56% for PFS. This is compared with 71% for OS and 69% for PFS in the GCB‐GEP group, and 82% for OS and 78% for PFS in the UC‐group (Figure 1A,D). Patients categorized as non‐GCB by the Hans algorithm showed inferior five‐year survival rates at 65% for OS and 62% for PFS, compared with 72% for OS and 71% for PFS in the GCB‐IHC group (Figure 1B,E). Patients grouped as ABC according to the Lymph2Cx assay and non‐GCB by the Hans algorithm demonstrated inferior five‐year survival rates at 53% for OS and 51% for PFS. This is compared with 74% for OS and 72% for PFS in cases that were not ABC and non‐GCB combined (Figure 1C,F). There were other variables associated with inferior OS (Table 2) and PFS (Table 3) in univariate analyses. They included age ≥ 60 years, B symptoms, ≥2 aaIPI, high stage (≥III), high LDH, extranodal involvement (only OS), high expression of MYC, BCL2 and double expression of MYC and BCL2.

**Table 2:** Univariate and multivariate Cox regression analyses with relative risk of overall survival (death due to any cause) estimated as hazard ratios with 95% confidence intervals and *P* values by putative prognostic factors in DLBCL patients. Statistical significance (*P* < .05) is indicated by boldface font

	Number of patients^a^	Univariate	Multivariate^b^
ABC (Lymph2Cx)^c^	271	**1.61:1.11‐2.36, .01**	1.71:0.95‐3.07, .07
Non‐GCB (Hans algorithm)^d^	349	1.36:0.97‐1.91, .07	1.53:0.92‐2.55, .10
Non‐GCB and ABC (Hans algorithm and Lymph2Cx)^e^	306	**2.00:1.39‐2.87, <.001**	**2.20:1.27‐3.81, .005**
Age ≥ 60 years	356	**3.16:2.00‐5.08, <.001**	Not included
Male	356	1.16:0.82‐1.63, .40	Not included
B symptoms	339	**1.77:1.26‐2.49, .001**	1.83:0.99‐3.39, .053
Age‐adjusted IPI ≥2	330	**2.00:1.42‐2.83, <.001**	1.04:0.56‐1.94, .90
Stage ≥III	337	**1.99:1.37‐2.88, <.001**	Not included
High LDH	341	**1.58:1.11‐2.24, .01**	Not included
Extranodal involvement	274	**1.55:1.07‐2.24, .02**	1.03:0.61‐1.73, .92
High expression of MYC	161	**1.91:1.16‐3.16, .01**	Not included
High expression of BCL2	212	**2.75:1.52‐5.00, <.001**	Not included
Double expression of MYC and BCL2	169	**2.57:1.51‐4.37, <.001**	**2.44:1.41‐4.21, .001**

Abbreviations: ABC, activated B‐cell; GCB, germinal center‐derived B‐cell; IPI, International Prognostic Index; LDH, Lactate Dehydrogenase.

*Note*: Boldface font indicates statistical significance (*P* < 0.05).

a Number of patients with information enabling evaluation of overall survival.

b ABC, Non‐GCB and Non‐GCB *and* ABC were included in separate multivariate models. Results for clinical and biologic variables are presented for the model where Non‐GCB was included. Variables of statistical significance (*P* < .05) or borderline statistical significance *(P* < .10) from the univariate analyses were included in the multivariate models. Since age ≥ 60 years, stage ≥III and high LDH are included in the age‐adjusted IPI ≥2 variable, and high expression of MYC and BCL2 are included in double expression of MYC and BCL2 variables, these variables were not included in the multivariate model.

c Compared with GCB‐GEP, UC cases were omitted from the analyses.

d Compared with GCB‐IHC.

e Compared with cases with information on both the Lymph2Cx assay and the Hans algorithm that were not ABC and non‐GCB combined.

**Table 3:** Univariate and multivariate Cox regression analyses with relative risk of progression‐free survival (disease progression or death due to any cause) estimated as hazard ratios with 95% confidence intervals and P‐values by putative prognostic factors in DLBCL patients. Statistical significance (*P* < .05) is indicated by boldface font

	Number of patients^a^	Univariate	Multivariate^b^
ABC (Lymph2Cx)^c^	269	**1.69:1.17‐2.45, .006**	1.65:0.92‐2.94, .09
Non‐GCB (Hans algorithm)^d^	347	**1.50:1.08‐2.08, .02**	**1.82:1.11‐2.98, .02**
Non‐GCB and ABC (Hans algorithm and Lymph2Cx)^e^	304	**2.04:1.42‐2.91, <.001**	**2.03:1.18‐3.50, .01**
Age ≥ 60 years	354	**2.79:1.78‐4.35, <.001**	Not included
Male	354	1.09:0.78‐1.51, .61	Not included
B symptoms	337	**1.73:1.25‐2.41, .001**	1.78:0.98‐3.24, .06
Age‐adjusted IPI ≥2	328	**2.12:1.51‐2.96, <.001**	1.16:0.63‐2.11, .60
Stage ≥III	335	**2.21:1.53‐3.18, <.001**	Not included
High LDH	339	**1.61:1.14‐2.26, .06**	Not included
Extranodal involvement	272	1.36:0.95‐1.96, .09	0.87:0.53‐1.46, .60
High expression of MYC	161	**1.87:1.14‐3.05, .01**	Not included
High expression of BCL2	210	**3.11:1.72‐5.63, <.001**	Not included
Double expression of MYC and BCL2	168	**2.59:1.54‐4.35 < .001**	**2.47:1.46‐4.19, <.001**

Abbreviations: ABC, activated B‐cell; GCB, germinal center‐derived B‐cell; IPI, International Prognostic Index; LDH, Lactate Dehydrogenase.

*Note*: Boldface font indicates statistical significance (*P* < 0.05).

a Number of patients with information enabling evaluation of progression‐free survival.

b ABC, Non‐GCB and Non‐GCB *and* ABC were included in separate multivariate models. Results for clinical and biologic variables are presented for the model where Non‐GCB was included. Variables of statistical significance (*P* < .05) or borderline statistical significance (*P* < .10) from the univariate analyses were included in the multivariate models. Since age ≥ 60 years, stage ≥III and high LDH are included in the age‐adjusted IPI ≥2 variable, and high expression of MYC and BCL2 are included in double expression of MYC and BCL2 variables, these variables were not included in the multivariate model.

c Compared with GCB‐GEP, UC cases were omitted from the analyses.

d Compared with GCB‐IHC.

e Compared with cases with information on both the Lymph2Cx assay and the Hans algorithm that were not ABC and non‐GCB combined.

### 3.4 Multivariate survival analysis

The different COO groups along with B symptoms, aaIPI ≥2, extranodal involvement and double expression of MYC and BCL2, were analyzed in multivariate Cox regression analyses. The COO determined by the Lymph2Cx assay was not associated with OS (Table 2) or PFS (Table 3). However, the non‐GCB patients according to the Hans algorithm had shorter PFS, represented by a hazard ratio (HR) of 1.82 (95% confidence interval [CI] 1.11‐2.98) (Table 3), but not OS (Table 2). In contrast, patients classified as ABC‐GEP by the Lymph2Cx assay and non‐GCB by the Hans algorithm displayed both shorter OS (HR = 2.20 [95% CI 1.27‐3.81]) (Table 2), and PFS (HR = 2.03 [95% CI 1.18‐3.50]) (Table 3). Double expression of MYC and BCL2 remained significantly associated with inferior OS and PFS in multivariate analysis. However, missing data for BCL2 and MYC was high: 41% and 55%, respectively (Table S4).

### 3.5 UC cases

Of 42 cases categorized as UC according to the Lymph2Cx assay, 33 (79%) were non‐GCB and 9 (21%) were GCB‐IHC according to the Hans algorithm (Table S3). There was a higher proportion of UC with a high expression of MUM1, and a lower proportion with a high expression of CD10, compared with GCB‐GEP and ABC cases according to the Lymph2Cx assay (data not shown). Although not statistically significant, tendencies were observed that a higher proportion of UC cases were younger (aged <60 years [*P* = .09]), and more often presented with B‐symptoms (*P* = .06) compared with GCB‐GEP and ABC cases. There were no major differences regarding double expression of MYC and BCL2 (Table S4). In supplementary analyses, UC patients showed no statistically significant associations with OS or PFS, in either univariate or in multivariate analysis (Tables S1 and S2).

## 4 DISCUSSION

Using GEP to determine the COO of DLBCL is undoubtedly the golden standard, but it requires fresh‐frozen material. Thus, it is also of great importance in order to determine the COO with high accuracy on FFPE material, since fresh‐frozen material is rarely available in clinical practice.^20^ NanoString technology with the application of the Lymph2Cx assay enables digital GEP on FFPE material, and we compared its concordance with the IHC algorithm by Hans et al in a large cohort of DLBCL patients from Sweden and Denmark homogenously treated with R‐CHOP. We report that the overall concordance between the Lymph2Cx assay, and the Hans algorithm was reasonable (72%). In more detail, GEP classified 85% of cases categorized as GCB by IHC as GCB, whereas only 58% classified as non‐GCB by IHC were ABC by GEP. This is partly because most of the UC cases according to the Lymph2Cx assay were non‐GCB by IHC (79%), and if the UC subgroup was omitted, GEP classified 75% of cases classified as non‐GCB by IHC as ABC. Our findings regarding concordance between IHC and GEP are largely in line with other studies.^10^, ^14^, ^16^, ^21^, ^22^ Several different IHC algorithms to determine COO have been proposed.^11^, ^12^, ^13^ The Hans algorithm uses CD10, BCL6 and MUM1, while the Choi scheme adds GCET1 and FOXP1 to the Hans algorithm.^12^ This has been associated with a slightly higher concordance with COO according to GEP than the Hans algorithm. Tally's algorithm uses LMO2 instead of BCL6 and was superior in determining COO compared to Hans' and Choi's algorithms in one study,^6^ while the Visco‐Young algorithm uses a five‐marker model of CD10, GCET1, FOXP1, MUM1, and BCL6.^13^ Nevertheless, no IHC algorithm will be 100% concordant with GEP regarding COO, and no IHC algorithm has so far been able to identify the UC cases. Thus, determining COO by GEP is the only way to identify the UC cases.

In our study, the survival outcome for the different GEP groups was well in concordance with previous studies^19^, ^22^, ^23^that also used the Lymph2Cx assay. Patients classified as ABC according to the Lymph2Cx assay had inferior OS and PFS in univariate analysis, but not in multivariate analysis. Patients classified as non‐GCB according to the Hans algorithm had inferior PFS, but were not associated with inferior OS in univariate or multivariate analyses. Notably, the best discriminator for survival was if cases were classified as non‐GCB/ABC‐GEP *both* by the IHC and the Lymph2Cx assays. This was significantly associated with inferior OS and PFS in both univariate and multivariate analyses, which has not been described in previous studies. These findings should be validated in other cohorts. Perhaps, cases at the extreme ends of the GCB and ABC spectrum are identified when COO is determined both by IHC and GEP. To determine COO with both IHC and GEP will result in an increased expense when patients with DLBCL are classified. However, in the era of precision medicine, more precise risk stratification is of utmost importance to identify patients that need intensified treatment regiments.^24^, ^25^, ^26^, ^27^, ^28^, ^29^, ^30^ Patients with the ABC subtype appear to have a greater benefit from targeted interventions, such as lenalidomide and bortezomib,via nuclear factor κB pathway inhibition, and ibrutinib, via Bruton's tyrosine kinase blockade.^31^ However, the preferential efficacy of these agents for the ABC group may be fully demonstrated when COO is determined with a robust technique.

The 2016 WHO classification recognizes and categorizes high‐grade B‐cell lymphomas (HGBCL) with *MYC* and *BCL2* and/or *BCL6* gene rearrangements as a separate entity, commonly referred to as “double‐hit” lymphomas. Whereas lymphomas with double expression of the proteins MYC and BCL2 are referred to as” double‐expressor lymphomas”.^24^, ^25^, ^26^, ^27^, ^28^, ^29^, ^30^ Patients with double gene rearrangements and double expression of MYC and BCL2 have an unfavorable prognosis and require intensified treatments. Patients with double gene rearrangements are more common in the GCB group, whereas double expressors are more common in the ABC group.^7^, ^25^, ^26^, ^32^ Since both double gene rearrangements and double expressors have inferior survival, the distribution of such cases may affect the outcome in both the ABC and GCB groups. Nonetheless, double‐hit lymphomas are infrequent (<10%), and are of limited prognostic impact in the GCB group. The patients in our study grouped as ABC both by IHC (non‐GCB) and the Lymph2Cx assay, were significantly more often both single and double expressors of MYC and BCL2. Both single MYC and BCL2 overexpression and double expression of MYC and BCL2 significantly affected survival outcome with an inferior outcome in univariate and multivariate analysis, which is in concordance with other studies.^7^, ^24^, ^25^, ^32^, ^33^

In multivariate survival analyses, non‐GCB/ABC according to both the Hans algorithm and the Lymph2Cx assay and double expression of MYC and BCL2 remained to be the two most robust discriminators of inferior outcome, while the clinical variables B symptoms, ≥2aaIPI and extranodal involvement failed to remain to be independent prognostic discriminators in multivariate analysis. Our findings indicate that COO and double expression of MYC and BCL2 are the most important factors in order to estimate survival outcome. This is also in line with other studies,^32^, ^34^ although both COO and double expression of MYC and BCL2 have not been independent prognostic factors in multivariate analysis in some previous studies.

Most (79%) of the UC cases according to the Lymph2Cx assay in our cohort belonged to the non‐GCB group according to the Hans algorithm. The UC cases in our study had an outcome comparable to patients classified as GCB. This is in contrast to other studies showing that the UC cases are more likely to be grouped together with the ABC subtype^10^, ^23^, ^35^ with an inferior patient outcome, although others have shown results similar to ours.^22^, ^33^, ^36^ The UC patients in our cohort did not differ significantly from GCB patients regarding clinical characteristics. Although, it appeared as if CD10 was less frequently expressed and MUM1 more common, whereas the expression of BCL6, MYC and BCL2 did not differ. The genetic composition of the obscure UC subgroup has remained largely unknown, however, a recent study found that concomitant *NOTCH2* mutations and *BCL6* translocations characterized the UC subgroup and were associated with a favorable survival outcome.^37^ These findings suggest that further subgrouping of ABC, GCB and UC, by including genetic data might be necessary in order to correctly risk stratify patients with DLBCL.

Our study was performed on a large cohort of DLBCL patients from Sweden and Denmark, where the characterization of COO has been performed both by IHC and GEP on FFPE material. The patient cohort in our study was not truly population‐based, but rather based on cases where enough tissue material was available, which may have caused a selection‐bias. Furthermore, in DLBCL patients whose tumors are located in deep anatomical sites where surgical biopsy is sparse are not included in most studies, which could add to the question of representativeness.^38^ However, clinical characteristics and survival were quite comparable to other studies of DLBCL patients.^8^, ^25^, ^39^, ^40^ We did not prepare new slides for the IHC stainings, but instead used the original ones performed by the primary laboratories. Still, the diagnoses and staining results were re‐evaluated and scored by the participating hematopathologists.

Our cases were classified according to the 2008 WHO classification, and very few were investigated for the presence of MYC, BCL2 and BCL6 rearrangements, which may represent a confounding bias. It was difficult to obtain material for the IHC stainings for MYC and BCL2, only half of the cases were investigated, but this is also observed in large prospective randomized multi‐center studies.^8^ Obtaining more material for FISH‐analyses would have been even more troublesome and would have selected cases with plentiful material. However, this is also the case for similar studies that included patients with HGBCL diagnosed prior to 2016. And, our study is no exception, and our cases have been reported as DLBCL according to the 2008 WHO classification, in order to make our study comparable with other studies. Presumably, some cases of HGBCL with MYC and BCL2 and/or BCL6 rearrangements may be included in our cohort. But, cases with “B‐cell lymphoma unclassifiable with features intermediate between diffuse large B‐cell lymphoma and Burkitt lymphoma”, according to the 2008 WHO classification, were not included. Many of the “double‐hit” lymphomas belong to this category, as does cases with transformation from previous follicular lymphoma, which were also excluded from our study. Thus, “double‐hit” lymphomas probably have limited impact on our results. Lastly, the primary aim with our study was to compare the utility of COO classification by the Lymph2Cx assay and IHC, according to the Hans algorithm of de novo DLBCL according to the 2008 WHO classification, not to study the prognostic impact of “double‐hit” lymphomas.

In conclusion, GEP combined with IHC to classify cases as ABC/non‐GCB is the best predictor of inferior survival, in both uni‐ and multivariate analyses, probably by identifying cases at the extreme ends of the GCB and ABC spectrum. We also found that cases classified by IHC as non‐GCB, were more often GCB‐GEP or UC than vice versa for the GCB‐IHC cases. Thus, IHC appears to be insufficient to identify cases of the ABC genotype. The Lymph2Cx assay is a robust assay that can be applicable on FFPE material in a clinical setting, in addition to conventional IHC, and is thus possible to implement on a routine clinical basis. Moreover, single as well as double expression of MYC and BCL2 significantly differed between ABC and GCB groups, which affected survival and may thus contribute to the dismal outcome for the ABC group. We propose that *both* GEP by the Lymph2Cx assay and IHC should be applied to determine COO in order to identify patients with the worst prognosis.

## CONFLICT OF INTEREST

The authors declare no conflicting interests.

## AUTHOR CONTRIBUTIONS

R.R., R.‐M.A., T.P., L.M. and L.C. designed and performed the study and M.A., P.H. and R.‐M.A. wrote the manuscript. M.A., P.H., T.P., L.M., S.B.E., P.‐O.A., M.H., M.F., M.E., S.D., H.M.P., F.A., K.G., G.E., L.C., R.R. and R.M.A. collected cases and performed analyses of data. M.A., S.B.E., M.H., M.F., H.M.P. and R.M.A. re‐evaluated the diagnoses and evaluated the IHC. All authors read and approved the final manuscript.

## Supporting information


**Supplementary Table S1** Cox regression analyses with relative risk of overall survival (death due to any cause) estimated as hazard ratios with 95% confidence intervals and P‐values among DLBCL patients. Statistical significance (*P* < .05) is indicated by boldface font.
**Supplementary Table S2.** Cox regression analyses with relative risk of progression‐free survival (disease progression or death due to any cause) estimated as hazard ratios with 95% confidence intervals and P‐values among DLBCL patients. Statistical significance (*P* < .05) is indicated by boldface font.
**Supplementary Table S3**. Association between the Lymph2Cx assay and the Hans algorithm.
**Supplementary Table S4.** Clinicopathological variables and their distribution in patients with UC and GCB‐GEP and ABC according to Lymph2Cx assay.
