# Supplementary material for: Cell‐of‐origin determined by both gene expression profiling and immunohistochemistry is the strongest predictor of survival in patients with diffuse large B‐cell lymphoma
Source: Am J Hematol. 2019 Nov 7;95(1):57–67. doi: 10.1002/ajh.25666 (PMC6916573; doi:10.1002/ajh.25666)
Supplement: Supplementary file 1 — Supplementary Table S1 Cox regression analyses with relative risk of overall survival (death due to any cause) estimated as hazard ratios with 95% confidence intervals and P‐values among DLBCL patients. Statistical significance (P < .05) is indicated by boldface font. Supplementary Table S2. Cox regression analyses with relative risk of progression‐free survival (disease progression or death due to any cause) estimated as hazard ratios with 95% confidence intervals and P‐values among DLBCL patients. Statistical significance (P < .05) is indicated by boldface font. Supplementary Table S3. Association between the Lymph2Cx assay and the Hans algorithm. Supplementary Table S4. Clinicopathological variables and their distribution in patients with UC and GCB‐GEP and ABC according to Lymph2Cx assay. [file AJH-95-57-s001.docx]

**Supplementary Methods**

In supplementary analyses, the following COO groups according to Lymph2Cx assay were studied: (1) the UC and GCB-GEP group compared with the ABC group; (2) the UC and ABC group compared with the GCB-GEP group; (3) the UC group compared with ABC and GCB-GEP group. For cases with information on both Hans algorithm and Lymph2Cx assay the following COO groups were studied: (1) GCB-IHC and GCB-GEP compared with cases that were non-GCB and/or ABC or UC; (2) non-GCB and ABC compared with GCB-IHC and GCB-GEP.

**Supplementary Table 1.** Cox regression analyses with relative risk of overall survival (death due to any cause) estimated as hazard ratios with 95% confidence intervals and P-values among DLBCL patients. Statistical significance (p < 0.05) is indicated by boldface font.

|  | Number of patients* | Univariate | Multivariate† |
| --- | --- | --- | --- |
| ABC *vs* GCB-GEP and UC | 312 | **1.67: 1.17-2.40, 0.005** | **1.76: 1.01-3.07, 0.045** |
| ABC and UC *vs* GCB-GEP | 312 | 1.37: 0.97: 1.95, 0.08 | 1.48: 0.87-2.50, 0.14 |
| UC *vs* ABC and GCB-GEP | 312 | 0.74: 0.43-1.27, 0.27 | 0.85: 0.40-1.81, 0.67 |
| GCB-GEP and GCB-IHC *vs* ABC, non-GCB and UC | 306 | 0.87: 0.61-1.25, 0.46 | 0.83: 0.49-1.41, 0.50 |
| ABC and non-GCB *vs* GCB-GEP and GCB-IHC‡ | 221 | **1.71: 1.15-2.54, 0.009** | 1.78: 0.97-3.27, 0.06 |

Abbreviations: ABC, Activated B-Cell; GCB, Germinal Center B-cell; UC, unclassified.

* Number of cases with information enabling evaluation of overall survival.

† For the multivariate models, B symptoms, age-adjusted IPI ≥2, extranodal involvement and double expression of MYC and BCL2 were included.

‡ABC and non-GCB versus GCB-GEP and GCB-IHC, other cases were omitted from the analyses.

**Supplementary Table 2.** Cox regression analyses with relative risk of progression-free survival (disease progression or death due to any cause) estimated as hazard ratios with 95% confidence intervals and P-values among DLBCL patients. Statistical significance (p < 0.05) is indicated by boldface font.

|  | Number of patients* | Univariate | Multivariate† |
| --- | --- | --- | --- |
| ABC *vs* GCB-GEP and UC | 310 | **1.72: 1.21-2.45, 0.003** | 1.68: 0.97-2.90, 0.07 |
| ABC and UC *vs* GCB-GEP | 310 | **1.44: 1.02-2.04, 0.04** | 1.46: 0.87-2.43, 0.15 |
| UC *vs* ABC and GCB-GEP | 310 | 0.78: 0.46-1.32, 0.35 | 0.92: 0.45-1.88, 0.82 |
| GCB-GEP and GCB-IHC *vs* ABC, non-GCB and UC | 304 | 0.80: 0.57-1.14, 0.23 | 0.70: 0.42-1.17, 0.17 |
| ABC and non-GCB *vs* GCB-GEP and GCB-IHC‡ | 220 | **1.83: 1.24-2.72, 0.003** | **1.93: 1.07-3.51, 0.03** |

Abbreviations: ABC, Activated B-Cell; GCB, Germinal Center B-cell.

* Number of cases with information enabling evaluation of progression-free survival.

† For the multivariate models, B symptoms, age-adjusted IPI ≥2, extranodal involvement and double expression of MYC and BCL2 were included.

‡ ABC and non-GCB versus GCB-GEP and GCB-IHC, other cases were omitted from the analyses.

**Supplementary Table 3**. Association between the Lymph2Cx assay and the Hans algorithm.

|  | ABC according to Lymph2Cx assay  (n = 105) | GCB-GEP according to Lymph2Cx assay  (n = 168) | UC according to Lymph2Cx  assay (n = 42) | P-value* |
| --- | --- | --- | --- | --- |
| Hans algorithm  Non-GCB (n = 151) (%)  GCB-IHC (n = 157) (%)  Missing (n = 7) (%) | 88 (58)  15 (9)  2 (29) | 30 (20)  133 (85)  5 (71) | 33 (22)  9 (6)  0 (0) | **<0.001** |

Abbreviations: ABC, activated B-cell; GCB, germinal center-derived B-cell; GEP, gene expression profiling; IHC, immunohistochemical; UC, unclassified

**Supplementary Table 4.** Clinicopathological variables and their distribution in patients with UC and GCB-GEP and ABC according to Lymph2Cx assay.

|  | UC  (%) | GCB-GEP and ABC (%) | p-value* |
| --- | --- | --- | --- |
| All patients | 42 (100) | 273 (100) |  |
| **Hans algorithm**  Non-GCB  GCB-IHC  Missing | 33 (79)  9 (21)  0 (0) | 118 (43)  148 (53)  7 (3) | **<0.001** |
| **Age**  Mean  Median  Range | 60  61  18-79 | 65  68  22-89 | **0.02**† |
| **Age ≥60 years**  Yes  No  Missing | 24 (57)  17 (40)  1 (2) | 197 (72)  74 (27)  2 (1) | 0.09 |
| **Male**  Yes  No  Missing | 27 (64)  15 (36)  0 (0) | 154 (56)  117 (43)  2 (1) | 0.46 |
| **B symptoms**  Yes  No  Missing | 21 (50)  18 (43)  3 (7) | 94 (34)  163 (60)  16 (6) | 0.06 |
| **≥2 age-adjusted IPI**  Yes  No  Missing | 23 (55)  16 (38)  3 (7) | 112 (41)  140 (51)  21 (8) | 0.13 |
| **Stage ≥III**  Yes  No  Missing | 29 (69)  11 (26)  2 (5) | 147 (54)  108 (40)  18 (7) | 0.11 |
| **High LDH**  Yes  No  Missing | 27 (64)  14 (33)  1 (2) | 139 (51)  118 (43)  16 (6) | 0.22 |
| **Extranodal involvement**  Yes  No  Missing | 13 (31)  19 (45)  10 (24) | 80 (29)  128 (47)  65 (24) | 0.97 |
| **High expression of MYC and BCL2**  Yes  No  Missing | 3 (7)  18 (43)  21 (50) | 28 (10)  97 (36)  148 (54) | 0.57 |

Abbreviations: ABC, Activated B-Cell; GCB, Germinal Center B-cell; IPI, International Prognostic Index; LDH, Lactate Dehydrogenase; UC, unclassified

*Comparing UC versus GCB-GEP and ABC according to Lymph2Cx assay.

†P-value according to Student’s t-test
